# Past as Prologue—Use of Rubella Vaccination Program Lessons to Inform COVID-19 Vaccination

**DOI:** 10.3201/eid2813.220604

**Published:** 2022-12

**Authors:** Meredith G. Dixon, Susan E. Reef, Laura A. Zimmerman, Gavin B. Grant

**Affiliations:** Centers for Disease Control and Prevention, Atlanta, Georgia, USA

**Keywords:** COVID-19, Rubella vaccination, coronavirus disease, SARS-CoV-2, severe acute respiratory syndrome coronavirus 2, viruses, respiratory infections, zoonoses, vaccine-preventable diseases

## Abstract

The rapid rollout of vaccines against COVID-19 as a key mitigation strategy to end the global pandemic might be informed by lessons learned from rubella vaccine implementation in response to the global rubella epidemic of 1963–1965. That rubella epidemic led to the development of a rubella vaccine that has been introduced in all but 21 countries worldwide and has led to elimination of rubella in 93 countries. Although widespread introduction and use of rubella vaccines was slower than that for COVID-19 vaccines, the process can provide valuable insights for the continued battle against COVID-19. Experiences from the rubella disease control program highlight the critical and evolving elements of a vaccination program, including clearly delineated goals and strategies, regular data-driven revisions to the program based on disease and vaccine safety surveillance, and evaluations to identify the vaccine most capable of achieving disease control targets.

As COVID-19 spreads throughout the world, we recall a similar experience of a swiftly spreading respiratory disease over half a century earlier. In 1963, a rubella virus epidemic spread from Europe to the United States, causing great alarm among public health officials. The New York Times reported on February 8, 1964: “GERMAN MEASLES AT EPIDEMIC RATE; City and State Affected—2,302 Cases Reported Here Since Dec. 1; Virus Is Termed Mild; But Women Are Warned of Danger During First 3 Months of Pregnancy” (*1*).

Although rubella is generally a mild disease, rubella infection during early pregnancy can be devastating. Fetal infection can result in miscarriage, stillbirth, or infants born with life-threatening or disabling congenital malformations, known as congenital rubella syndrome (CRS). A pregnant woman infected with rubella in early pregnancy has up to a 90% chance of giving birth to an infant with CRS and that infant having >1 malformations, such as congenital heart defect, cataracts, and hearing impairment. CRS is the most substantial public health threat of rubella infection and is associated with an infant mortality rate of 20%–40% and lifelong sequelae for many of those infants that survive (*2*).

The outbreak of rubella in 1963 necessitated expeditious development of a vaccine to protect pregnant women and their infants and to stem societal disruption from the subsequent epidemic. Later, licensure and widespread availability of vaccines prevented future epidemics of rubella in the United States and other countries. As of October 2021, a total of 173 (89%) of 194 countries have introduced rubella vaccine, and 93 (48%) have been declared free of endemic rubella transmission (*3*).

Rubella and SARS-CoV-2 viruses have several similarities (Table 1). Both viruses are enveloped, positive-stranded RNA viruses (*2*,*4*) that are transmissible through respiratory droplets. Both viruses can result in asymptomatic infections, fostering silent disease transmission (*2*,*5*). On average, in countries with no available rubella vaccine, 1 rubella-infected person can infect 6–12 other susceptible persons (*6*), an infection rate similar to that of the SARS-CoV-2 Delta and Omicron variants (*7*,*8*). Both viruses are associated with serious disease complications. For rubella, the most serious complication is CRS (*2*); COVID-19 complications include respiratory failure, multisystem inflammatory syndromes, post—COVID-19 conditions, and preterm delivery or stillbirth (*9*,*10*). Both viral infections can result in death. Like deaths attributed to rubella infection before vaccine introduction, most COVID-19–related deaths occur in specific high-risk populations. SARS-CoV-2 infections cause higher mortality among the elderly and those with specific underlying conditions than among younger, generally healthy adults. Because of the commonalities of SARS-CoV-2 and rubella, the rubella disease control program might serve as a useful comparator in formulating COVID-19 vaccination strategy and implementation.

**Table 1:** Comparison of rubella and SARS-CoV-2 viruses*

Comparator	Rubella	SARS-CoV-2
Type of virus	Enveloped, positive-stranded RNA virus	Enveloped, positive-stranded RNA virus
Virus classification	Rubivirus in Matonaviridae family	Coronavirus in Coronaviridae family
Reservoir	Humans only	Mainly birds and mammals
Subtypes	1 serotype	Numerous variants with continual evolution
Transmission	Mainly respiratory droplet	Mainly respiratory droplet
Incubation period range, d	12–23	1–14
Reproductive number	6–12	6–10
Nature of clinical manifestations	Asymptomatic through mild prodromal symptoms to miscarriage and stillbirth	Asymptomatic to severe illness
Infections that are asymptomatic, %	20–50	31–40
Serious complications	Congenital rubella syndrome	Respiratory failure, multisystem inflammatory syndromes, post–COVID-19 conditions, stillbirths and preterm births
Major risk factors for serious complications	Infection early in pregnancy increases likelihood of CRS	Age, certain underlying medical conditions
Vaccine efficacy against infection, %	97	90
Waning immunity after vaccination	Seropositivity rates ranged 92%–100% 1–21 y after 1 dose	Possible; vaccine efficacy/effectiveness rates decreased on average 21 percentage points 1–6 mo after final vaccine dose of primary series, although mechanism not fully elucidated and multiple limitations exist

*CRS, congenital rubella syndrome.

We believe that the US rubella disease control program, which incorporated strategic planning, goal communication, program initiation, and program revisions driven by data, provides key insights for developing vaccines to combat the COVID-19 pandemic. Here, we highlight key components of the rubella disease control and elimination program, including vaccination strategies and vaccine selection methods, and describe how these experiences might inform current COVID-19 vaccination programs.

## Vaccination Strategy

The primary goal of a vaccination program is to reduce disease burden by achieving high population immunity levels through strategies aimed at both optimal immunization coverage and high vaccine effectiveness. The success of vaccination strategies depends greatly on practical aspects of implementation. An individual protection, or selective, approach targets specific groups that are defined by such factors as risk or age. The aim of this approach is to protect vulnerable groups against disease and severe outcomes (hospitalization, complications, death). Although the individual protection approach can prevent severe outcomes, some high-risk persons can be missed. In instances when the entire population is at risk of infection, there is ongoing transmission risk to vulnerable persons. Thus, a universal approach might be a better strategy in such circumstances since this approach indirectly impacts vulnerable subgroups by increasing population-level immunity and potentially interrupting or even eliminating virus transmission. A universal approach requires vaccines with high efficacy against infection across a wide range of vaccine recipients and viral subtypes.

The primary goal of national rubella vaccination programs is to prevent rubella infection in pregnant women and thereby prevent the severe outcome of CRS. When rubella emerged as a nationwide threat, 2 vaccination strategies were implemented to achieve this goal: an individual protection approach that prioritized vaccinating high-risk populations (adolescent females and women of childbearing age) to prevent CRS; and a universal approach that aimed to decrease and interrupt transmission at the population level by vaccinating the age group with the highest proportion of susceptible persons: primarily, young children and those potentially at highest risk (e.g., reproductive-age women).

In 1970, the United Kingdom adopted the individual protection approach, primarily vaccinating nonpregnant women of childbearing age. This decision was informed by concerns at that time regarding unknown duration of vaccine-induced immunity in children, as well as the fact that measles vaccination coverage in the United Kingdom was low and rubella vaccine would have been given at the same time as the measles vaccine (*11*). Surveillance data showed that this approach decreased the incidence of CRS cases and termination of pregnancies associated with rubella (*12*). However, because the approach only focused on individual protection, viral transmission continued in the population at large. Unvaccinated women in the United Kingdom continued to be infected, and children continued to be born with CRS, albeit at a lower rate (*11*). Studies demonstrated that unprotected persons still posed a risk. For example, in 1 study, pregnant women with previous pregnancies had a higher risk of rubella infection than did women in their first pregnancies, suggesting that women with previous pregnancies may have been at risk of acquired rubella infection from their own children with rubella (*13*). Whereas control of rubella through individual protection was proving to be inadequate, immunization program advancements had occurred, and measles vaccination coverage had increased, which prompted UK policymakers to pivot to the universal approach: vaccinating all young children to protect the larger population.

In contrast to the initial UK approach, the United States launched its rubella vaccination program in 1969, using the universal approach. Children >1 year of age up to puberty were vaccinated against rubella with the aim of eliminating rubella virus transmission and infection. From 1969 through 1977, an estimated 80 million doses of rubella virus vaccine were distributed in the United States (*14*). As in the United Kingdom, the rubella vaccination program was systematically monitored through disease surveillance, seroprevalence studies, and vaccination coverage assessments (*15*). Those data illustrated that susceptibility remained high among women of childbearing age and that they were still being exposed. To decrease the rubella immunity gaps resulting from the universal approach, which was focused on pediatric vaccination, the United States expanded its rubella vaccination strategy in 1978 to include vaccination of older groups. After this policy shift and through the late 1980s, cases of rubella infection and CRS in the United States declined further (*16*). We provide a comparison of the individual protection and universal strategies (Table 2).

**Table 2:** Comparison of the 2 strategies used for rubella control and elimination activities*

Comparator	Individual protection strategy	Universal strategy
Strategic target	High-risk individuals	Susceptible population
Populations	Women of child-bearing age	Infants and campaigns targeting susceptible individuals
Initial goals	Reduce cases of CRS	Elimination of rubella and CRS
Strategy used when	Low infant vaccination coverage; concerns for safety	High infant vaccination coverage (>80%)
Monitoring systems	Surveillance for CRS; rubella vaccination coverage; special surveys/studies	Surveillance for rubella and CRS; rubella vaccination coverage; special surveys and studies
Examples	Initial United Kingdom strategy; initial global (WHO) strategy	Initial United States strategy; current global (WHO) strategy

*CRS, congenital rubella syndrome; WHO, World Health Organization.

By determining disease burden and monitoring vaccination impact, disease control experts used rubella and CRS surveillance data to iteratively inform rubella disease control strategy and used vaccination coverage data to determine the progress of vaccination programs. Additional activities (e.g., monitoring vaccine safety through pregnancy registries, adverse events surveillance) provided data to ensure vaccine safety and gain public confidence. Those data sources were critical in determining the progress of specific programs. In the United States, surveillance data documented an end to rubella outbreaks by autochthonous transmission, and elimination was verified in 2004. In the United Kingdom, rubella surveillance and vaccination program data prompted a change in program strategy to a universal protection approach, which led to elimination, verified in 2016. The United States and the United Kingdom still continue to experience imported rubella cases from countries with high levels of ongoing transmission, usually from countries with low immunization coverage or those that have not introduced rubella vaccine (*16*). As such, both countries would still benefit from global elimination.

Globally, the World Health Organization (WHO) initially recommended an individual protection approach to rubella disease control, which evolved to a universal strategy. The first WHO recommendation in 2000 focused on ensuring that women of childbearing age were protected, without preference for a specific strategy (*17*). By 2011, WHO recommended both the individual protection and the universal strategies for countries, with a preference for the universal approach (*18*). In 2020, the WHO position shifted to recommending only the universal approach (*2*).

Although global strategies have shifted over time in response to new data, inequities in global rubella program implementation have been evident in both introduction and elimination activities. Introduction was initially only in high-income countries, but by 2020, rubella vaccine had been introduced in 48% of low-income countries. Of 21 countries that had not introduced rubella vaccine by the end of 2020, a total of 14 were low-income countries (*19*). Of the 93 countries that have eliminated rubella disease, only 3 were identified as low-income countries.

## Vaccine Selection

The 1964–1965 rubella epidemic resulted in an estimated 12.5 million rubella cases in the United States, infecting 6% of the US population. Complications included >2,000 cases of encephalitis, 11,350 cases of miscarriage, and 20,000 cases of CRS. Of the CRS cases, >8,000 children were diagnosed with deafness, 3,580 were diagnosed as blind, and 1,800 children had developmental delays. The total estimated economic impact was $1.5 billion (*20*). 

The epidemic catalyzed rubella vaccine development, which incorporated new laboratory techniques that, in turn, allowed the quick isolation of the rubella virus. During 1969 and 1970, a total of 4 rubella vaccines were licensed, 3 in the United States (HPV77-DE, HPV-77-DK, Cendehill) and 1 in Europe (RA27/3) (*21*). Each vaccine was administered as a single dose that provoked a durable, protective immune response when given to a person >9 months of age. 

The 4 rubella vaccines were studied continuously for both effectiveness and safety. Immunogenicity of each rubella vaccine was studied from multiple perspectives. HPV77-DE was implemented widely in the United States and found to be immunogenic in 95% of vaccinees and protected 65%–94% of recipients during outbreaks. In contrast, RA27/3 achieved seroconversion in 95%–100% of vaccine recipients (*22**,**23*); in numerous outbreaks, protection from RA27/3 was >95% (*6*). Antibody levels in 8 comparative studies demonstrated that RA27/3 generated 2- to 4-fold higher antibody levels than either the Cendehill or HPV-77 vaccine (*22*). Furthermore, compared with the Cendehill vaccine, the RA27/3 vaccine produced higher antibody levels 6–8 weeks after vaccination (*22*). Later studies demonstrated that such antibody response to RA27/3 persisted many years after receipt of the vaccine (*22*). Challenge studies have shown that when vaccinated persons were exposed to wild rubella virus, only 3%–10% of the RA27/3 vaccine recipients experienced reinfection (i.e., had breakthrough infections) compared with 40%–100% of the HPV-77 or Cendehill vaccine recipients (*22*).

Research also evaluated and compared the safety of these vaccines. The RA27/3 vaccine provoked lower rates of adverse reactions among adults than did HPV-77-DK or HPV-77-DE, both of which were associated with significant acute joint reactions (*22*). The safety of vaccination during pregnancy, especially in regard to vaccine-associated CRS, was a chief concern for disease control experts and limited vaccination strategies initially employed in the United States (*24*). However, evidence slowly accumulated, including from mass vaccination campaigns in the Americas, that provided strong evidence that rubella vaccine did not cause CRS (*25*).

The higher effectiveness of the RA 27/3 vaccine, coupled with lower rates of adverse reactions, led to the vaccine’s widespread adoption as the preferred rubella vaccine in the United States, resulting in its licensure in 1979 and the withdrawal of HPV-77 and Cendehill vaccines (*23*). Additional surveillance and comparative research studies strengthened the RA 27/3 vaccine’s status as being especially effective in eliciting a strong immune response, decreasing risk of rubella virus transmission, and achieving these results with a very favorable safety profile (*21*). These findings resulted in this vaccine being accepted and used in almost all countries. A systematic literature review in 2019 showed that both single-dose and 2-dose regimens of rubella vaccine are highly immunogenic for a long period of time (*26*).

## Lessons Learned from Rubella Vaccination in the COVID-19 Context

Today, rubella transmission has been eliminated in many countries throughout the world as a result of data-driven strategies and an effective, highly immunogenic, and safe vaccine that was developed and approved over time through rigorous scientific research and surveillance. The success of rubella control and elimination as we have described might inform policymakers as they make decisions regarding the COVID-19 vaccine program.

As was the case for the rubella pandemic, the COVID-19 pandemic has resulted in the rapid development and deployment of multiple vaccines. Unlike rubella virus, which had infected persons prior to its pandemic spread, SARS-CoV-2 emerged as a new virus to which the entire global population was susceptible. Although the rubella vaccine has yet to be introduced in 21 countries, COVID-19 vaccines have been introduced in every country (*27*). Vaccination inequities do, however, exist for COVID-19 vaccine introduction and use. High-income countries have achieved higher coverage than middle- and low-income countries. Limited vaccine supply, insufficient immunization program capacity, and socioeconomic issues have contributed to this disparity in regard to global vaccination (*28*).

When the highly constrained supply of the first COVID-19 vaccines became available in late 2020, COVID-19 vaccination followed an individual protection approach, focusing on protecting the highest-risk populations and then expanding eligibility as vaccine supplies grew. Much like the United Kingdom’s initial individual protection approach to rubella vaccination that resulted in a substantial decline in CRS cases, this selective approach resulted in sharp declines in COVID-19 hospitalizations and deaths among the vaccinated but left the unvaccinated at risk of infection and serious disease (*29**,**30*). Now that more COVID-19 vaccines have been approved and the vaccine supply expanded, vaccination has been broadened to a larger pool of eligible persons. This increased supply, coupled with new and ever-growing knowledge of each vaccine’s advantages and disadvantages, has further informed COVID-19 vaccination program goals and efforts. In addition, surveillance measures have helped to identify priority populations for COVID-19 vaccination and monitor progress toward risk mitigation and population recovery. Vaccine safety surveillance systems and clinical studies have provided vital information to identify vaccine-associated adverse events.

As the COVID-19 vaccine supply increases and the pandemic evolves, comparative studies with objective criteria are needed to identify the vaccine(s) that meet the immediate goals of the global COVID-19 vaccination strategy, which is to minimize deaths, severe disease, and overall disease burden; curtail the health system impact; fully resume socio-economic activities; and reduce the risk of new variants (*31*). Currently available COVID-19 vaccines must be closely examined to distinguish which are most efficient in providing high seroconversion rates, long-term immunity against infection, serious illness, hospitalization, and death, and low rates of adverse events. The challenge of finding an optimal COVID-19 vaccine is compounded given that, unlike the rubella virus, which has only 1 serotype and no variants, SARS-CoV-2 variants continue to emerge (*4*). Ongoing COVID-19 vaccine effectiveness protocols and studies provide critical data that help researchers better understand troublesome trends, such as waning of vaccine-induced immunity or variant immune evasion (*32*–*34*), which further inform vaccine development and vaccination program goals. Innovative studies that examine varying vaccine schedules and combinations are underway, which will help to identify not only the most ideal vaccines but possibly also the best combination of vaccines (*33*).

Beyond vaccination strategies and vaccine choices, governments and public health authorities must consider other factors to help meet COVID-19 control goals. In terms of program implementation, key elements include cost considerations, expiration timeframes, cold chain requirements, and storage capacity. From a community perspective, factors affecting vaccination include preferences regarding administration and delivery, access to health services, and trust in healthcare providers and government information. Epidemiologically informed policy and control goals, when clearly and effectively communicated by trusted and empathetic sources, can create a unified vision for how nations can collectively bring an end to the COVID-19 pandemic, while proactively countering disinformation.

Disease control goals and vaccination strategy go hand in hand. Adopting a universal approach, as clinical trial data and licensure permit, would ensure that the world population can benefit from COVID-19 vaccination. Such an approach would require policymakers to address structural barriers to ensure access, equity, and confidence in vaccination. Thus, the success of achieving the goal of disease control depends on fully implementing the accompanying consensus strategy.

## Conclusions

The success of the rubella disease control program provides valuable insights for the continuing battle against COVID-19. Key elements to a successful program include clearly delineated goals and strategies, regular data-driven revisions to the program based on surveillance, safety, and epidemiologic data, and evaluations to identify the most appropriate vaccine(s) to achieve disease control targets. Comparative vaccine studies are necessary to help identify the most appropriate vaccine to achieve programmatic goals, especially given the increase in both assortment and supply of COVID-19 vaccines. Whereas data guide strategic decision-making in determining a global response to the COVID-19 pandemic, such other factors as vaccine confidence and equity play important roles in defining and clearly communicating the programmatic goals. Those goals, at present, include protecting individuals from severe disease, hospitalization, and death as well as reducing health system strain and limiting emergence of new variants.
